# Heterointerface Effect in Accelerating the Cathodic Oxygen Reduction for Intermediate-Temperature Solid Oxide Fuel Cells

**DOI:** 10.3389/fchem.2022.959863

**Published:** 2022-08-16

**Authors:** Yu Meng, Xiaofei Zhu, Jiao Meng, Jinghe Bai, Ruyi Chen, Defeng Zhou, Ning Wang, Dan Tian

**Affiliations:** ^1^ School of Chemistry and Life Science, Changchun University of Technology, Changchun, China; ^2^ Tangshan Caofeidian District Caofeidian New Town Experimental School (Beijing Jingshan School Caofeidian Branch), Tangshan, China; ^3^ Tangshan Industry Vocational Technology College, Tanghai Caofeidian Campus, Tangshan, China; ^4^ Shenzhen Institute of Advanced Electronic Materials, Shenzhen Institute of Advanced Technology, Chinese Academy of Sciences, Shenzhen, China; ^5^ College of Materials Science and Engineering, Nanjing Forestry University, Nanjing, China

**Keywords:** solid oxide fuel cell, composite cathode, heterointerface, oxygen vacancy, electrocatalytic activity

## Abstract

A solid-state mixing method was adopted to prepare a new Pr_0.8_Sr_0.2_Fe_0.7_Ni_0.3_O_3−δ_-Pr_1.2_Sr_0.8_Fe_0.4_Ni_0.6_O_4+δ_ (PSFN_113-214_) composite cathode oxide for the solid oxide fuel cells (SOFCs). Herein, heterointerface engineering was investigated for the performance enhancement. It was found that the oxygen vacancy content could be increased by mixing the PSFN_214_ with PSFN_113_, which gave rise to the formation of a heterostructure, and resulted in the promotion of oxygen ion transport as well as the specific surface area. The optimum mixing ratio 5:5 resulted in the highest oxygen vacancy content and the largest specific surface area, indicating the strongest interface effect. Polarization resistance of PSFN_113-214_ (5:5) was 0.029 Ω cm^2^ at 800°C, which was merely 24% of PSFN_113_ and 39% of PSFN_214_. The corresponding maximum power density was 0.699 W cm^−2^, which was nearly 1.44 times of PSFN_113_ and 1.24 times of PSFN_214_. Furthermore, the voltage attenuation rate after 100 h was merely 0.0352% h^−1^. Therefore, the new PSFN_113-214_ composite could be a prospective cathode oxide for SOFCs.

## Introduction

The development of intermediate-temperature solid oxide fuel cells (IT-SOFCs) is severely limited because of its restricted cathodic oxygen reduction reaction (ORR) rate (Huan et al., 2021; Ahmad et al., 2022). Therefore, high-performance cathode materials have become a research hotspot (Ajaa et al., 2020).

Perovskite oxides possess extraordinary conductivity but poor surface mobility. Ruddlesden-Popper (R-P) phase oxides possess a special structure of alternating layers of perovskite and rock salt, which provides more oxygen vacancies as well as oxygen gaps. Unfortunately, their conductivity is relatively lower (Liu et al., 2016; Lee and Lee, 2017; Yu et al., 2019). Hence, it is impossible for single-phase materials to fully satisfy the conditions of cathode materials. Heterostructure cathode materials possess sufficient contact sites between oxygen and heterointerfaces, which can not only enhance electronic or ionic conductivity but also have potential to enhance stability and catalytic performance. It has thus become a hot research area for IT-SOFCs cathode materials. Studies have shown significantly enhanced electrochemical performance of heterogeneous composite cathodes compared to single-phase materials, as seen in La_0.5_Sr_0.5_CoO_3-δ_-LaSrCoO_4±δ_ (Li et al., 2020), La_0.6_Sr_0.4_Co_0.2_Fe_0.8_O_3−δ_-La_0.6_Sr_1.4_Co_0.2_Fe_0.8_O_4−δ_ (Wang et al., 2020), La_0.6_Sr_0.4_Co_0.2_Fe_0.8_O_3−δ_-La_2_NiO_4+δ_ (Dong et al., 2019; Ghamarinia et al., 2020), PrSrFe_0.5_Co_0.5_O_4_-Pr_0.4_Sr_0.6_Fe_0.5_Co_0.5_O_3_ (Yu et al., 2019), and Nd_0.5_Sr_0.5_CoO_3−δ_-Nd_0.8_Sr_1.2_CoO_4±δ_ (Zheng et al., 2019; Zheng et al., 2020a; Zheng et al., 2020b). However, cobalt-based materials have some disadvantages, such as poor chemical stability, high price, and high coefficient of thermal expansion. Consequently, Cobalt-free ABO_3_-A_2_BO_4_ heterocomposite cathodes come into our sight. For example, the introduction of Sr ions into Pr_2_Ni_0.5_Mn_0.5_O_4−δ_ could form a PrO_x_-(PrSr)Ni_0.5_Mn_0.5_O_3−δ_-(PrSr)_2_(MnNi)O_4−δ_ heterocomposite cathode. Among them, the heterostructure formed at the interface of (PrSr)Ni_0.5_Mn_0.5_O_3−δ_ and (PrSr)_2_(MnNi)O_4−δ_ could improve the electrochemical performance, and the maximum power density (PPD) enhanced from 483 to 960 mW cm^−2^ at 800°C (Yang et al., 2019). Introducing La_0.5_Sr_1.5_MnO_4+δ_ into La_0.5_Sr_0.5_MnO_3−δ_ to form La_0.5_Sr_1.5_MnO_4+δ_-La_0.5_Sr_0.5_MnO_3−δ_, the ORR would be broadened to entire cathode region, and PPD was 936 mW cm^−2^ at 700°C (Hou et al., 2020). Porous (La_0.6_Sr_0.4_)_0.98_FeO_3−δ_ electrodes impregnated with aqueous nitrate solutions of Pr_2_Ni_0.6_Cu_0.4_O_4_ and Pr_2_Ni_0.7_Cu_0.3_O_4_ greatly reduced the polarization resistance, from 0.98 Ω cm^2^ to 0.13 Ω cm^2^ and 0.16 Ω cm^2^ at 650°C (Khoshkalam et al., 2020).

Among ABO_3_ perovskite materials, Pr_1−x_Sr_x_Fe_1−y_Ni_y_O_3−δ_ has broad application prospects due to its outstanding electrocatalytic activity and excellent electrical conductivity (Hashimoto et al., 2005; Larramendi et al., 2007; Pinedo et al., 2011; Giuliano et al., 2017; Liu et al., 2017). Especially, the conductivity of the Pr_0.7_Sr_0.3_Fe_0.7_Ni_0.3_O_3−δ_ cathode at 600°C was up to 450 S cm^−1^, and the electrochemical performance was similar to La_0.6_Sr_0.4_Fe_0.8_Co_0.2_O_3−δ_ (Hashimoto et al., 2005). Cathode materials containing Sr are prone to segregation of SrO during cell operation. Therefore, in the preparation process of ABO_3_ material, we could reduce the content of Sr as much as possible to obtain a more stable cathode material. Pr_0.8_Sr_0.2_Fe_0.7_Ni_0.3_O_3−δ_ cathode reduced Rp by only 6% within 200 h, and was stable within 1000 h (Giuliano et al., 2017). In A_2_BO_4_ materials, Ln_2_NiO_4+δ_ (Ln = La, Pr, Nd) material is the most widely studied cathode material. Pr_2_NiO_4_ (PNO) had the lowest polarization resistance and the highest oxygen surface exchange coefficient (k*) and diffusion coefficient (D*) therein (Bansod et al., 2018; Kim et al., 2019). However, its thermal stability was poor, and it was easy to decompose during operation. The structure stability is improved by doping Sr^2+^ at the Pr site (Bhoga et al., 2014; Kim and Lee, 2021). Among Pr_2−x_Sr_x_NiO_4_ (x = 0.3, 0.5, and 0.8) cathode materials, Pr_1.2_Sr_0.8_NiO_4_ had the lowest area-specific resistance value when the Sr doping amount was 0.8, down to 0.112 Ω cm^2^ at 800°C (Yang et al., 2012). In addition, the substitution of Ni sites with Fe can increase the oxygen surface exchange and show excellent performance. For example, in the La_1.5_Sr_0.5_Ni_1−y_Fe_y_O_4+δ_ series, iron doping promoted the bulk diffusion of the sample, and the oxygen surface exchange was significantly increased when y = 0.4 (Gilev et al., 2018). La_1.2_Sr_0.8_Ni_0.6_Fe_0.4_O_4+δ_ had the performance suitable for IT-SOFCs cathode. Its polarization resistance (Rp) was 0.078 Ω cm^2^, and its PPD was up to 781 mW cm^−2^ at 700°C (Miao et al., 2019). Therefore, this study chose Pr_0.8_Sr_0.2_Fe_0.7_Ni_0.3_O_3−δ_ (PSFN_113_) and Pr_1.2_Sr_0.8_Fe_0.4_Ni_0.6_O_4+δ_ (PSFN_214_) as the two components of the composite material. The structure, compatibility, microstructure, specific surface area, and electrochemical activity of single-phase materials and heterogeneous composite cathode materials were studied comparatively, and their potential as IT-SOFCs was evaluated.

## Experimental

### Chemicals

The chemicals utilized in this study, including Pr(NO_3_)_3_·6H_2_O (Aladdin, 99.9%), Sr(NO_3_)_2_ (Aladdin, 99%), Fe(NO_3_)_3_·9H_2_O (Aladdin, 98.5%) and Ni(CH_3_COO)_2_·6H_2_O (Aladdin, 99.9%), are used as received without further purification.

### Preparation of Cathode Materials

Single-phase Pr_0.8_Sr_0.2_Fe_0.7_Ni_0.3_O_3−δ_ (PSFN_113_) and Pr_1.2_Sr_0.8_Fe_0.4_Ni_0.6_O_4+δ_ (PSFN_214_) cathode powders were synthesized via the sol-gel method. First, citric acid was completely dissolved in distilled water under stirring. Raw materials including Pr(NO_3_)_3_·6H_2_O (Aladdin, 99.9%), Sr(NO_3_)_2_ (Aladdin, 99%), Fe(NO_3_)_3_·9H_2_O (Aladdin, 98.5%), and Ni(CH_3_COO)_2_·6H_2_O (Aladdin, 99.9%) were joined in sequence in corresponding proportions. The proportion of citric acid was twice than of metal ions. Subsequently, 2 g of polyethylene glycol powder was added. After stirring for 2–3 h, the above solution was dehydrated in a water bath at 80°C for 12 h to gain a dry gel. This dry gel was heated on a hot plate to form a precursor powder. After sufficient grinding, the precursor powder was calcined in a muffle furnace at 600°C for 4 h. Ultimately, this pre-fired powder was ground and calcined in a muffle furnace at 900°C for 4 h to obtain the desired cathode material.

Pr_0.8_Sr_0.2_Fe_0.7_Ni_0.3_O_3-δ_-Pr_1.2_Sr_0.8_Fe_0.4_Ni_0.6_O_4+δ_ (PSFN_113-214_) heterogeneous composite cathode was synthesized via solid-state mixing method, in which mass ratio of PSFN_113_ and PSFN_214_ was 4:6, 5:5 and 6:4 respectively. First, PSFN_113_ powder was poured into ethanol solvent according to the corresponding proportion, and zirconia ball was milled to make it evenly distributed. Subsequently, PSFN_214_ powder of the corresponding quality was poured into it and ball milled for 24 h. Above-mentioned mixture solution was put in an oven and dried continuously at 80°C for 12 h to gain dry powder. Finally, the gained powder was ground in a mortar for 48 h to gain a uniformly mixed composite cathode.

### Cell Construction

The symmetrical cell was assembled as follows. First, Ce_0.8_Gd_0.2_O_1.9_ (GDC) powder was pressed at 10 MPa into a disc with a diameter of 13 mm and a thickness of 1 mm. A compact GDC electrolyte sheet was formed *via* sintering at 1500°C for 5 h in muffle furnace. Then, 6wt% terpineol/ethyl cellulose binder was mixed with PSFN_113_, PSFN_214_, PSFN_113-214_ heterogeneous composite cathodes, and the required five kinds of cathode slurries were obtained after the grinding for several hours. Cathode slurry was symmetrically coated on both sides of the Ce_0.8_Gd_0.2_O_1.9_ (GDC) electrolyte by screen printing, calcined in a muffle furnace at 900°C for 2 h. Ultimately, the collector silver slurry was symmetrically coated in the grid structure on both sides of the electrolyte, and dried in an oven at 200°C for 2 h.

Similarly, a single cell was assembled. The GDC electrolyte sheet was polished to ∼250 μm and ultrasonically cleaned to obtain the required electrolyte sheet. NiO-GDC anode was prepared by mixing NiO and GDC at a mass ratio of 6:4. The required PSFN_113_, PSFN_214_, PSFN_113-214_ (5:5) cathode slurry, and NiO-GDC anode slurry were constructed as described above. First, NiO-GDC anode paste was screen printed on one side of the GDC electrolyte. After calcination at 1250°C for 4 h, the cathode slurry was symmetrically coated on the other side of the electrolyte. After calcinating at 900°C for 2 h, current collector silver paste was coated on both sides of the cell to form a grid structure.

### Characterization

An X-ray diffractometer with a Cu Kα X-ray source (*λ* = 0.15406 nm, 40 kV, 200 mA) was used to collect the X-ray diffraction (XRD) data from 20° to 80°, so as to determine the purity, thermal stability, crystal structure and chemical compatibility of the synthetic powder. Fourier infrared spectrometer was utilized to characterize the functional group structure of the synthesized powder in the range of 400–4000 cm^−1^. A field emission scanning electron microscope equipped with an X-ray spectrometer (EDS) was utilized to observe the cathode powder morphology and cathode/electrolyte interface adhesion. A high-resolution transmission electron microscope was utilized to further verify the existence of a heterointerface. Under the condition of degassing at 180°C for 12 h, a rapid specific surface area analyzer was utilized to measure the specific surface area of the material. X-ray photoelectron spectrometer was utilized to determine the oxygen vacancy content on the surface and valence state change.

### Electrochemical Test

The symmetrical cell was tested by electrochemical impedance spectroscopy (EIS) at 500–800°C using an electrochemical workstation. The current-voltage (I-V) and current-power density (I-P) curves of the single-cell were measured at 500–800°C using the SI 1287 electrochemical interface. Ultimately, the long-term stability of the single-cell was evaluated under the condition of a constant current density of 0.3 A cm^−2^ at 700°C.

## Results and Discussion

### Phase Analysis

**FIGURE 1:**
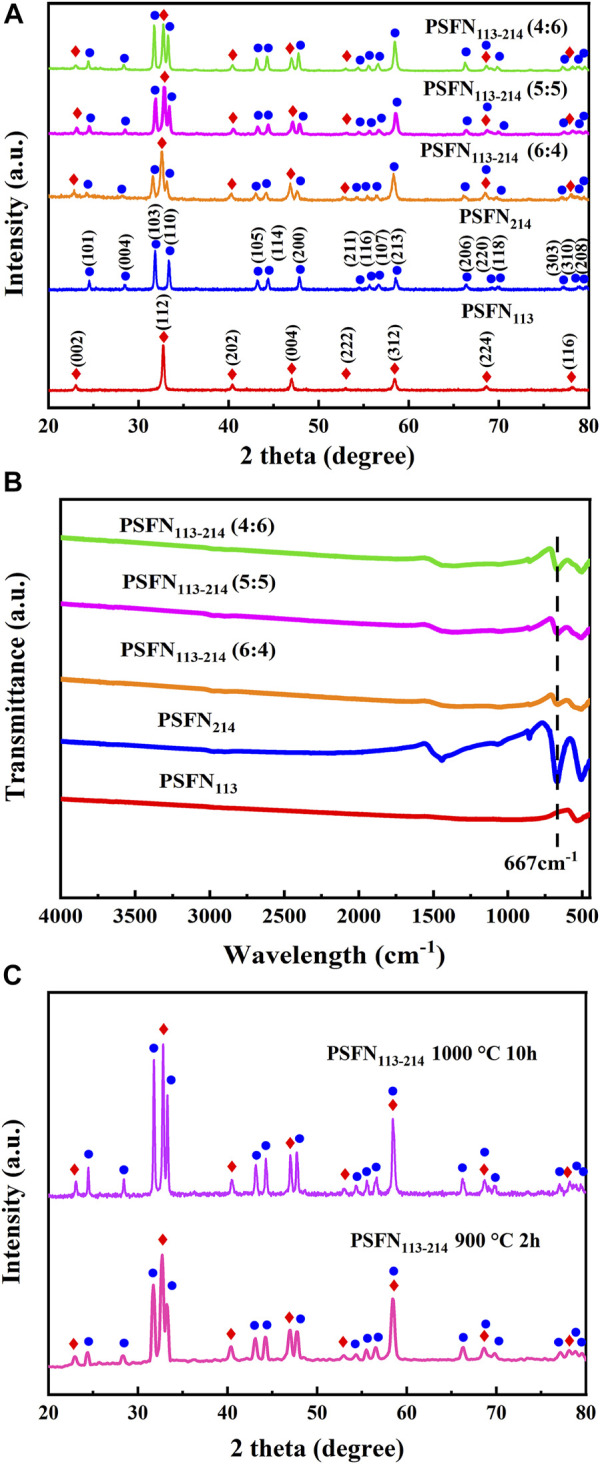
**(A)** XRD of cathode powder; **(B)** infrared spectrum of cathode powder; **(C)** XRD of PSFN_113-214_ mixed in equal proportion at different calcination temperatures.

To study the structure and phase purity of obtained cathode powder, XRD tests are performed on PSFN_113_, PSFN_214,_ and PSFN_113-214_, as shown in Figure 1A. PSFN_113_ powder showed a good orthorhombic perovskite structure with a space group of Pbnm, which matched well with the crystal structure of La_0.7_Sr_0.3_Co_0.5_Fe_0.5_O_3_ (PDF#89-1267) (Ajaa et al., 2020). PSFN_214_ had a typical K_2_NiF_4_ type tetragonal structure with a space group of I4/mmm, which matched well with the crystal structure of Sr_2_FeO_4_ (PDF#82-0414) (Giuliano et al., 2017; Miao et al., 2019). The diffraction peaks of PSFN_113-214_ heterogeneous composites were entirely in accordance with the above-mentioned two single-phase cathode, and no other impurity peaks were observed. In addition, corresponding characteristic peak intensity raised little by little with the increase of PSFN_214_ mass ratio, indicating that three different proportions of heterogeneous composite cathodes had been synthesized.

To further verify the (PSFN_113_:PSFN_214_) ratios of heterogeneous composite oxides, Fourier transforms infrared spectroscopy (FT-IR) tests are performed on all samples. Unlike simple ABO_3_ perovskites, A_2_BO_4_ consists of alternating perovskite (ABO_3_) and salt rock formations (AO) in the c-axis direction. Therefore, A_2_BO_4_ contains the characteristic peaks of ABO_3_ and AO formations. As shown in Figure 1B, the absorption peak intensity of the AO formation at 667 cm^−1^ gradually increased in proportion to the increase of PSFN_214_ content. It showed that the heterogeneous composite material had been mixed uniformly and satisfied the expected ratio change, which was consistent with the above-mentioned XRD peak intensity change.

To determine the stability of the PSFN_113-214_ heterogeneous composite cathode material, we mixed the quality mass of PSFN_113_ and PSFN_214_. After calcination at 900°C for 2 h and 1000°C for 10 h, XRD test is performed, as shown in Figure 1C. These results showed that the PSFN_113-214_ heterogeneous composite cathode still maintained its single-phase structure without any change in composition, which met the requirements of long-term operation.

### Chemical Compatibility

**FIGURE 2:**
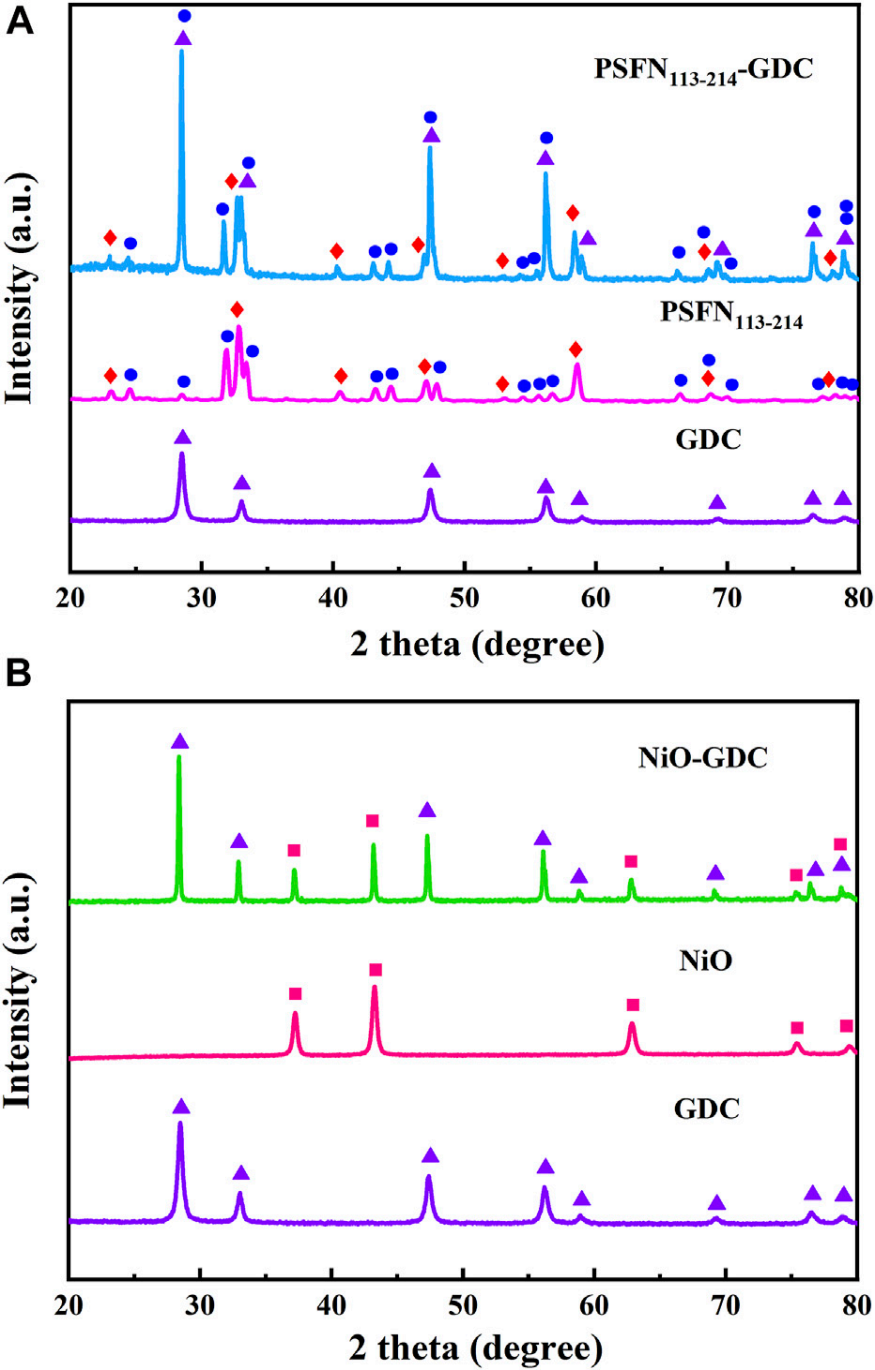
After calcination at 1000°C for 10 h, **(A)** XRD patterns of PSFN_113-214_ and GDC mixed in equal proportions and **(B)** XRD patterns of NiO and GDC mixed in equal proportions.

In addition, the long-term stable operation of the cell is affected *via* chemical compatibility of the electrode and the electrolyte (Zhang et al., 2021). Therefore, PSFN_113-214_ (5:5) and GDC electrolyte oxides were mixed with equal mass in an ethanol medium, and the compatibility of the two was tested by calcination at 1000°C for 10 h, as shown in Figures 2A,B. XRD pattern only contained the characteristic peaks of the PSFN_113-214_ cathode/NiO anode component and the GDC electrolyte, and no other impurity peaks are detected. This showed that the electrode and the electrolyte existed stably with each other during the calcination process and could be used for long-term operation.

### Microstructure Analysis

**FIGURE 3:**
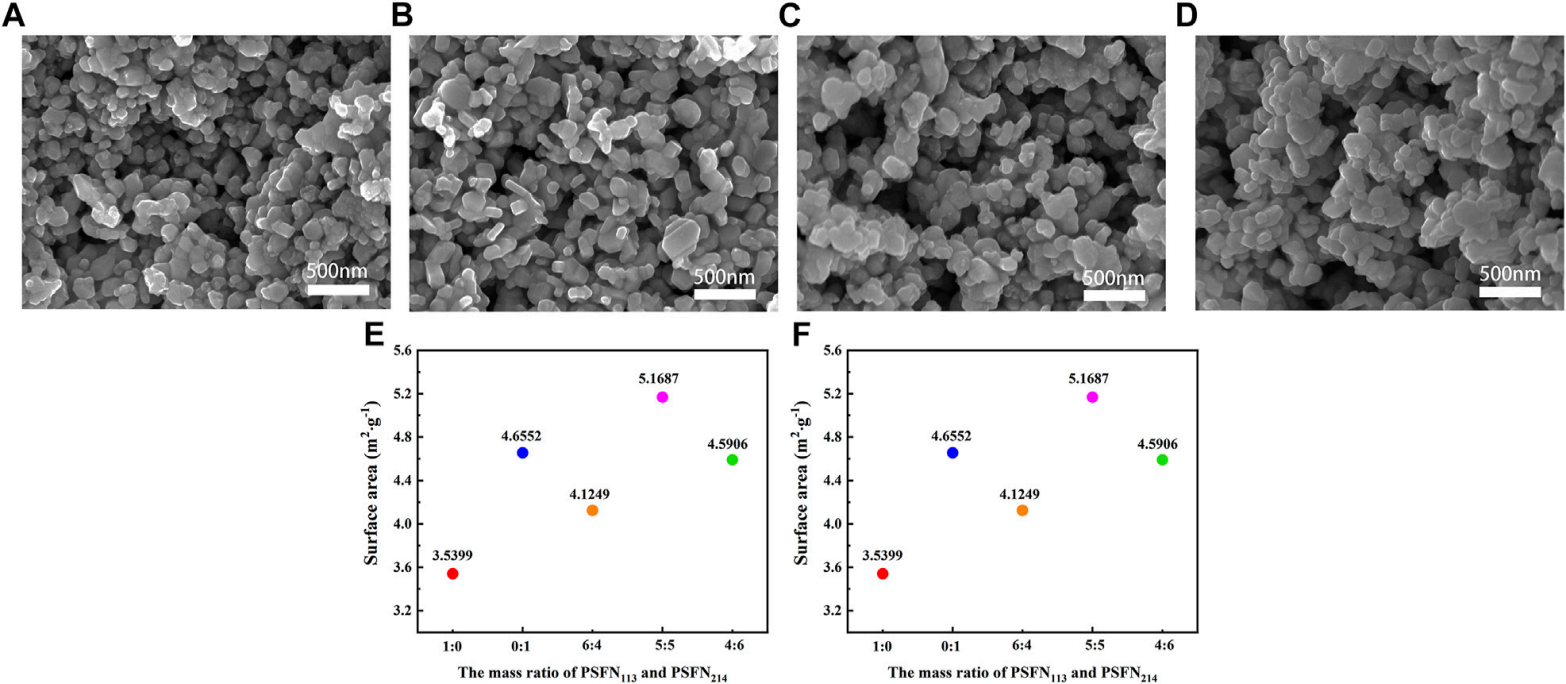
FE-SEM images of **(A)** PSFN_113_, **(B)** PSNF_214_, **(C)** PSFN_113-214_ (6:4), **(D)** PSFN_113-214_ (5:5), and **(E)** PSFN_113-214_ (4:6) cathode microstructure. **(F)** Specific surface area of all samples.

Electrode microstructure is one of the important factors affecting cell performance, which is closely related to material porosity and the three-phase interface (TPB) area (Han et al., 2021). The microstructure of the cathode material is shown in Figure 3A–E. All samples had a small and uniform particle size, good connectivity between the two-phase particles, and a porous structure. The porous structure facilitates gas diffusion and oxygen ion transport and also provides adequately active sites for ORR. In addition, heterocomposite cathode had no obvious bidirectional characteristics in appearance, indicating that the two single phases constituting the composite cathode were fully mixed and tightly wound together.

To further clarify the microstructure of cathode material, N_2_ adsorption-desorption test is carried out, as shown in Figure 3F. The results showed that the specific surface area of the five samples with mass ratios of PSFN_113_ and PSFN_214_ of 1:0, 0:1, 6:4, 5:5, 4:6 was 3.5399 m^2^ g^−1^ and 4.6552 m^2^ g^−1^ 4.1249 m^2^ g^−1^, 5.1687 m^2^ g^−1^ and 4.5906 m^2^ g^−1^, respectively. It can be seen that when PSFN_113_ and PSFN_214_ particles were tightly wound to form a heterointerface, the specific surface area of the composite cathode oxides was greater than PSFN_113_, and the corresponding ORR reaction active sites were increased. When the mass ratio of PSFN_113_ to PSFN_214_ was 5:5, the specific surface area value was the largest, which was expected to have the best electrochemical performance.

### Surface Chemical Environmental Analysis

**FIGURE 4:**
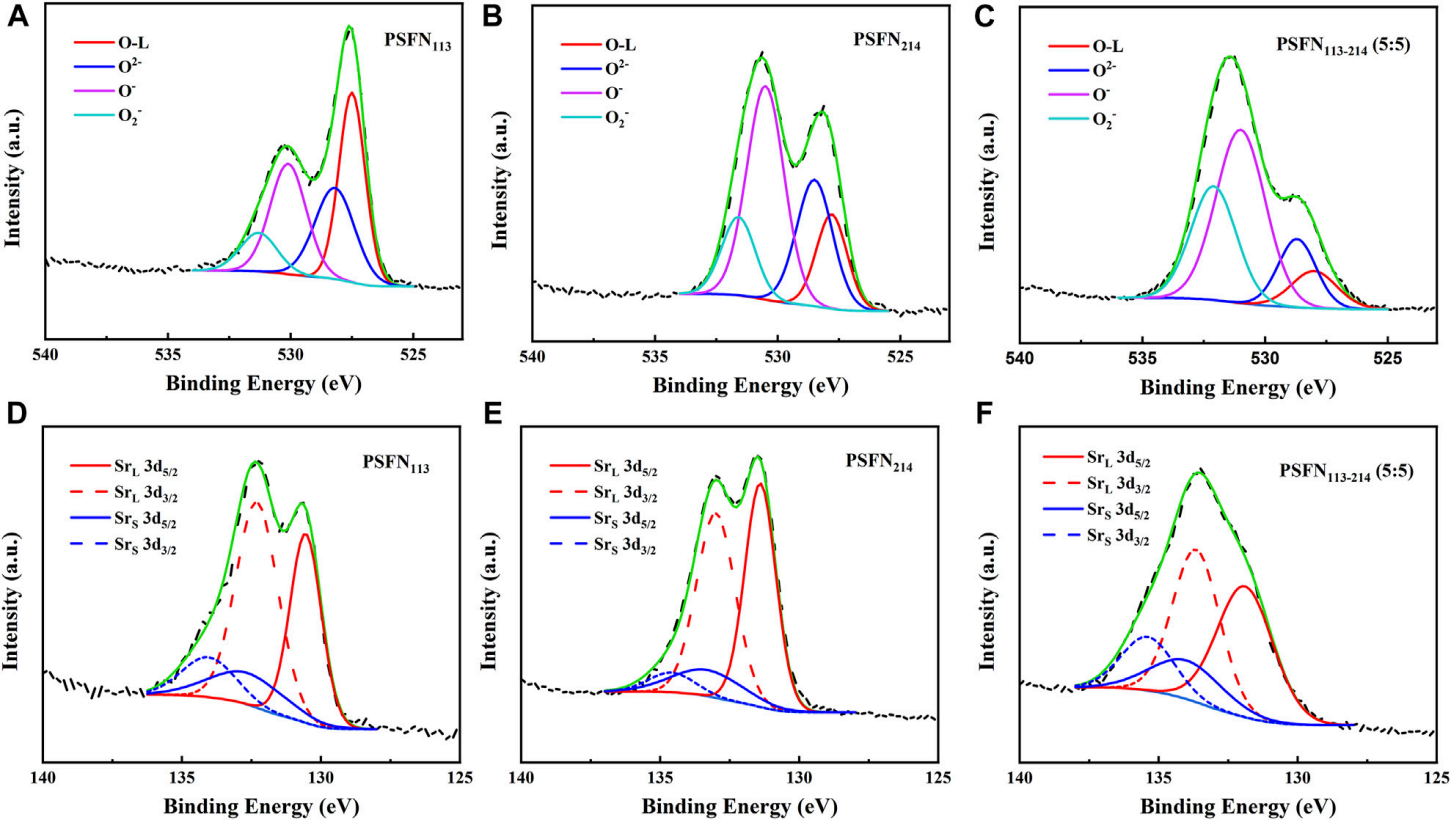
XPS spectra of PSFN_113_, PSFN_214_ and PSFN_113-214_ (5:5) cathode materials obtained for **(A–C)** O1s and **(D–F)** Sr 3d.

To further investigate the influence of the presence of heterointerfaces on the oxygen reduction catalytic activity and structural stability of the cathode material, the orbitals of O 1s and Sr 3d were collected by XPS, as shown in Figure 4. Previous studies have shown that the O element can be separated into lattice oxygen (O_lattice_, ∼528.5 eV) and surface oxygen (O_surface_) (Wang H. et al., 2019). Surface oxygen includes three types: O^2−^ (∼529.6 eV), O^−^ (∼531.1 eV), and ${\text{O}}_{2}^{-}$ (∼532.4 eV) (Bai et al., 2021). Among them, the content of O_surface_ ( ${\text{O}}_{2}^{-}$ O^−^) mainly affects the surface oxygen vacancies on the cathode and the ORR activity (Wang J. et al., 2019; Bai et al., 2021). So, the higher the content, the stronger the ORR catalytic activity. As shown in Table 1, the presence of heterointerface increased O_surface_ from 64.61% (PSFN_113_) and 83.96% (PSFN_214_) to 90.61% (PSFN_113-214_). It can be concluded that the existence of the heterointerface has a beneficial influence on the catalytic activity of oxygen reduction.

**TABLE 1:** The percentage of O_surface_, O_lattic_e, Sr_surface_, and Sr_lattice_ in PSFN_113_, PSFN_214_, and PSFN_113-214_ (5:5).

Cathode	O_surface_ (%)	Sr_surface_ (%)	Sr_lattice_ (%)
PSFN_113_	64.61	56.42	43.59
PSFN_214_	83.96	47.84	52.16
PSFN_113-214_	90.61	49.88	50.12

Similarly, Sr elements can be separated into lattice strontium (Sr_lattice_) and 1Q (Sr_surface_). Sr_lattice_ in perovskite lattices have less binding energies (∼131.7 eV, 3d_5/2_, ∼133.5 eV, 3d_3/2_) (Wang H. et al., 2019). Sr_surface_ on perovskite surfaces has large binding energies (∼133.8 eV, 3d_5/2_; ∼135.2 eV, 3d_3/2_), such as Sr(OH)_2_, SrCO_3,_ or SrO barrier layers (Bai et al., 2021). The higher the surface strontium content, the greater the degree of segregation of the Sr element, which will impair the electrical activity of the cell. Therefore, the less the content of Sr_surface_, the better the stability of the cathode (Wang J. et al., 2019; Bai et al., 2021). As shown in Table 1, the presence of heterointerfaces neutralized Sr_surface_ from 56.42% (PSFN_113_) and 47.84% (PSFN_214_) to 49.88% (PSFN_113-214_). It can be concluded that the existence of the heterointerface has a neutralizing effect on the structural stability. Whether it can meet the requirements for long-term operation of cathode oxides will be confirmed in the follow-up long-term stability test.

### High-Resolution Transmission Electron Microscope Analysis

**FIGURE 5:**
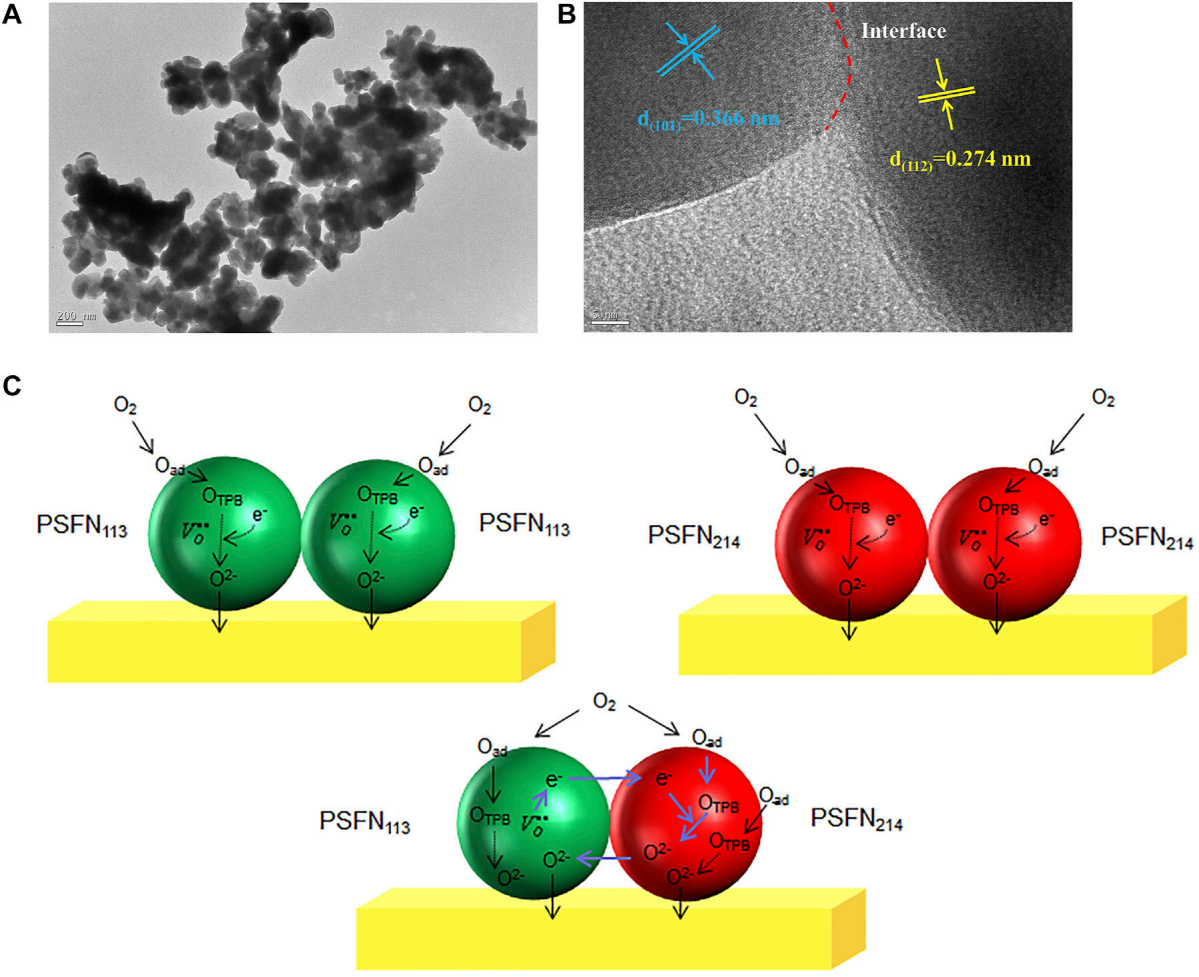
**(A)** TEM image, **(B)** HR-TEM image, and **(C)** performance improvement mechanism diagram of PSFN_113-214_ (5:5) heterocomposite cathode.

To determine the micromorphology of the heterocomposite cathode material in one step, a high-resolution transmission electron microscope (HR-TEM) is utilized to observe the micromorphology of the PSFN_113-214_ (5:5) heterocomposite cathode, as shown in Figures 5A,B. It can be seen from Figure 5A that the PSFN_113-214_ heterocomposite cathode particles were tightly entangled and had a good contact area. Two distinct diffraction fringes can be observed in Figure 5B. The diffraction fringe with a pitch of 0.274 nm matched the orthogonal (112) plane of PSFN_113_, while the diffraction fringe with a pitch of 0.366 nm matched the (101) plane of the tetragonal phase of PSFN_214_ (Yu et al., 2019). There was an obvious interface between PSFN_113_ and PSFN_214_ nanoparticles. The electrons in PSFN_113_ could be transferred to PSFN_214_ through the heterointerface, and the oxygen ions in PSFN_214_ could also be transferred to PSFN_113_ through the heterogeneous interface. This synergistic effect of the two was expected to promote the catalytic activity of oxygen reduction. The above mechanism is shown in Figure 5C.

### Electrochemical Impedance Spectroscopy Spectra

**FIGURE 6:**
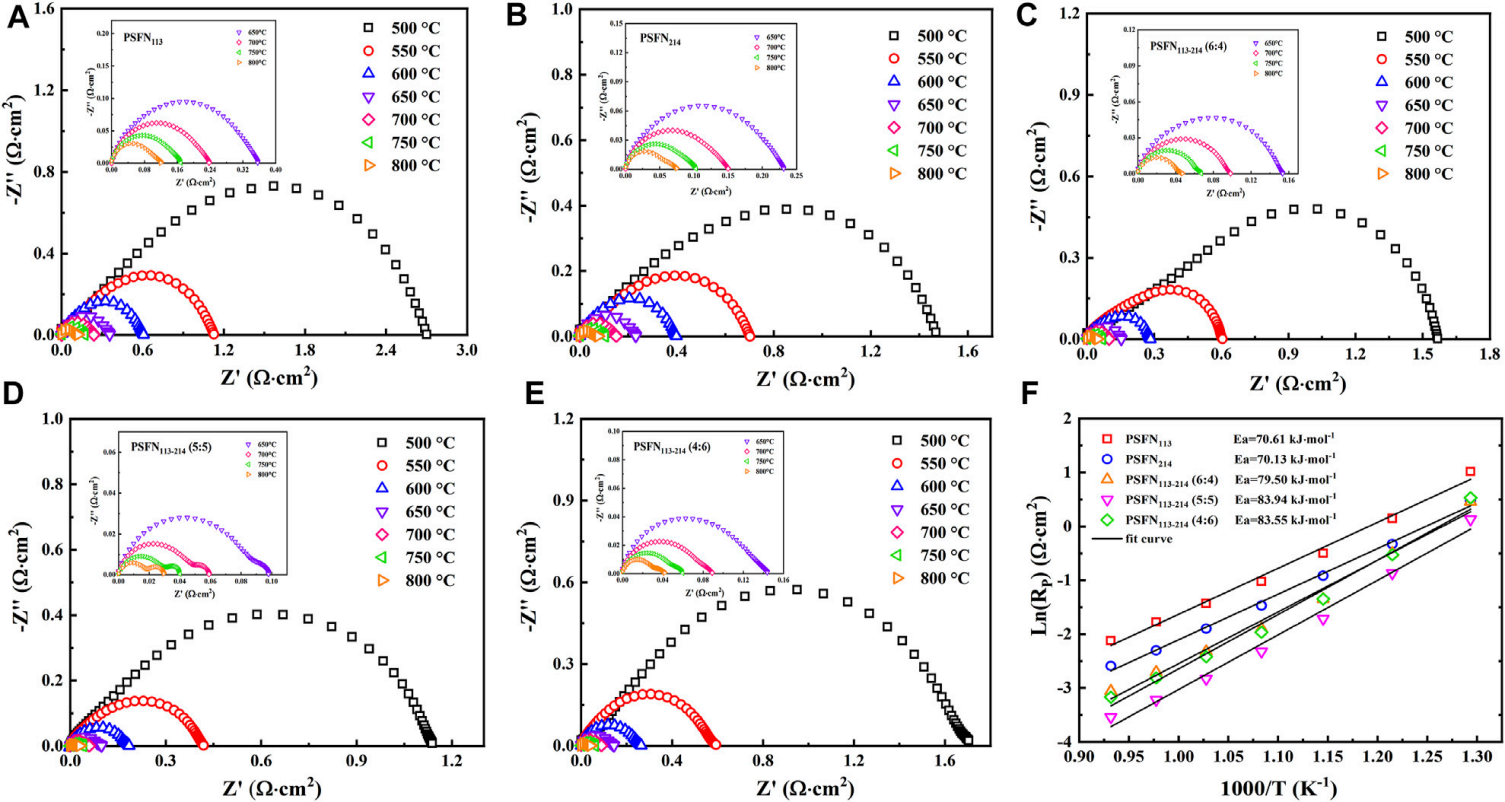
Electrochemical impedance spectrum of a symmetrical cell: **(A)** PSFN_113_, **(B)** PSFN_214_, **(C)** PSFN_113-214_ (6:4), **(D)** PSFN_113-214_ (5:5), **(E)** PSFN_113-214_ (4:6), and **(F)** polarization resistance varies Arrhenius curve of temperature change.

To research the effect of the presence of heterointerface on electrochemical activity, we constructed five symmetrical cells with mass ratios of PSFN_113_ and PSFN_214_ of 1:0, 0:1, 6:4, 5:5, and 4:6. At 500–800°C, the EIS test is performed on three symmetrical batteries at intervals of 50°C, as shown in Figures 6A–E. The cathodic polarization resistance (Rp) is the difference between the real axis intercepts of the impedance diagram (Li et al., 2017), and the corresponding values are shown in Table 2. Within the above temperature range, the R_P_ of the three materials all followed the following sequence: PSFN_113-214_ (5:5)< PSFN_113-214_ (4:6)<PSFN_113-214_ (6:4)<PSFN_214_ < PSFN_113_. It showed that the existence of heterointerface would promote the cathodic oxygen reduction reaction, and the catalytic activity of PSFN_113-214_ oxygen reduction was significantly enhanced, which greatly improved the performance of IT-SOFCs (Zhao et al., 2019). Among them, the PSFN_113-214_ (5:5) cathode exhibited the lowest polarization resistance. Its R_P_ value (0.029 Ω cm^2^) at 800°C was only 24% of PSFN_113_ (0.12 Ω cm^2^) and 39% of PSNF_214_ (0.075 Ω cm^2^). It can be seen that the heterointerface of PSFN_113-214_ (5:5) could be maximized, which was consistent with the above-mentioned specific surface area analysis and oxygen vacancy content. To compare the R_P_ changes of the three samples more intuitively, we made the Arrhenius curve of R_P_ and temperature, as shown in Figure 6F. As shown in the figure, the activation energy of PSFN_113-214_ heterocomposite cathode was greater than PSFN_113_ (74.65 kJ mol^−1^) and PSFN_214_ (74.16 kJ mol^−1^), indicating that the catalytic activity of the composite oxide was more sensitive to temperature (Li et al., 2017; Kuzmin et al., 2020), but still much smaller than La_0.5_Sr_0.5_CoO_3−δ_-LaSrCoO_4±δ_ (121.25 kJ mol^−1^) (Li et al., 2020), (PrSr)Ni_0.5_Mn_0.5_O_3−δ_-PrO_x_-(PrSr)_2_(MnNi) O_4−δ_ (147.5 kJ mol^−1^) (Wang et al., 2020). Therefore, PSFN_113-214_ (5:5) has the potential as a SOFC cathode material.

**TABLE 2:** Rp values of PSFN_113_ and PSFN_214_ cathode oxides with different mass ratios.

Temperature/°C	1:0 R_P_/Ω·cm^2^	0:1 R_P_/Ω·cm^2^	6:4 R_P_/Ω·cm^2^	5:5 R_P_/Ω·cm^2^	4:6 R_P_/Ω·cm^2^
800	0.12	0.075	0.047	0.029	0.042
750	0.17	0.10	0.067	0.040	0.060
700	0.24	0.15	0.098	0.059	0.089
650	0.36	0.23	0.15	0.098	0.14
600	0.61	0.40	0.26	0.18	0.26
550	1.16	0.72	0.61	0.42	0.59
500	2.77	1.61	1.58	1.14	1.70

### Interface Microstructures

**FIGURE 7:**
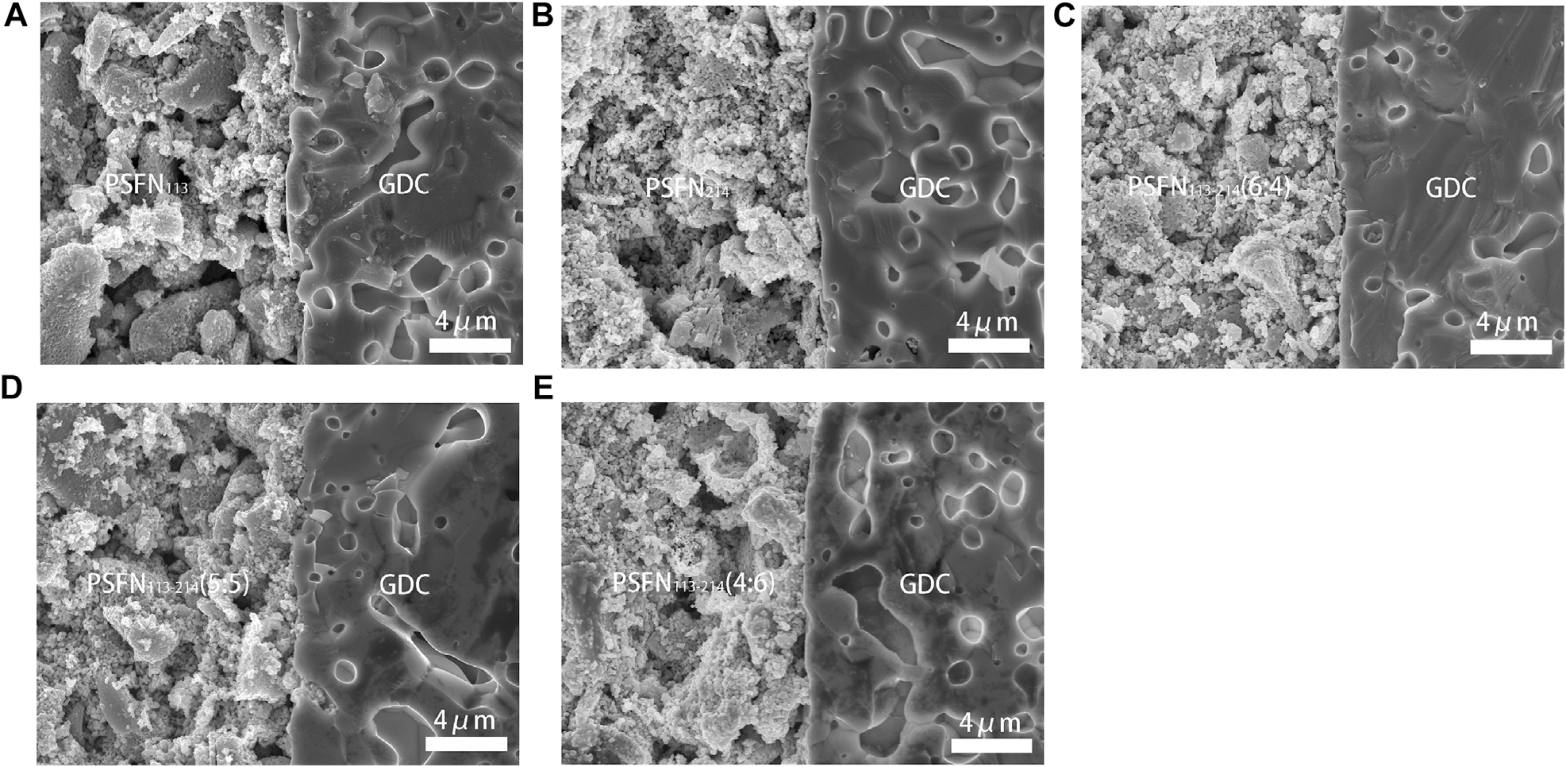
After the EIS test, the cross-sectional SEM of the symmetrical cell with **(A)** PSFN_113_, **(B)** PSFN_214_, **(C)** PSFN_113-214_ (6:4), **(D)** PSFN_113-214_ (5:5), and **(E)** PSFN_113-214_ (4:6) as the cathode.

To research the thermal compatibility of cathode material and electrolyte material in symmetrical cells, the cross-section FE-SEM of cathode and electrolyte is observed, as shown in Figure 7. It can be seen from the figure that both the electrolyte GDC and the cathode material had a clear interface. Particles were evenly distributed and very tightly attached to the GDC electrolyte, without obvious delamination and cracks, which was conducive to gas transmission and oxygen diffusion.

### Single Cell Performance

**FIGURE 8:**
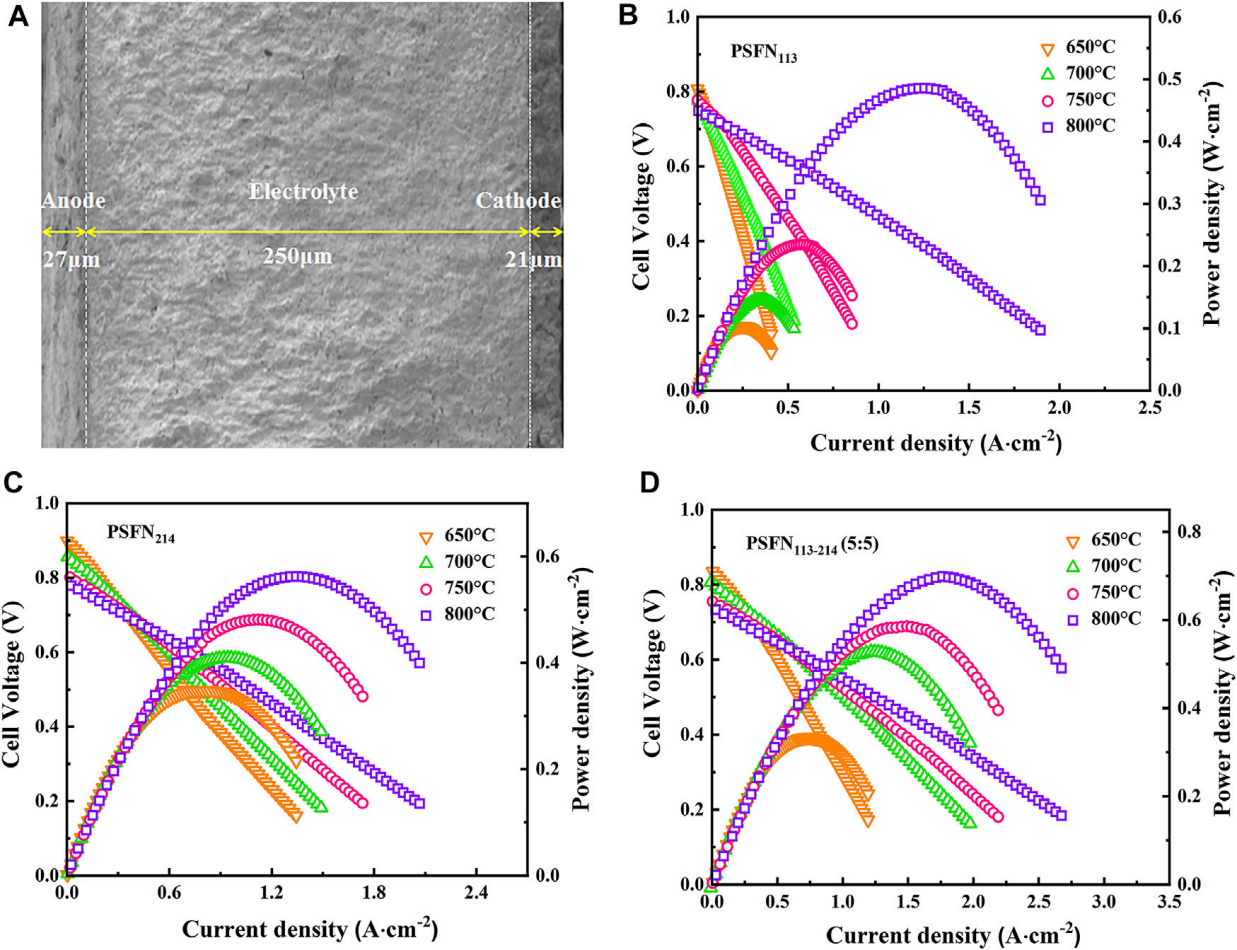
**(A)** Cross-sectional morphology of a single cell and I-V-P curves: **(B)** PSFN_113_, **(C)** PSFN_214_, and **(D)** PSFN_113-214_ (5:5).

To further evaluate the effect of the heterointerface on the cathodic oxygen reduction reaction, an electrolyte-supported NiO-GDC/GDC/[PSFN_113_/PSFN_214_/PSFN_113-214_ (5:5)] single cell was constructed, denoted as Cell-I, Cell-II, and Cell-III. Figure 8A represents the cross-sectional morphology of a single cell. As shown in the figure, the thickness of the GDC electrolyte was about 250 μm, and the thickness of the cathode and anode was between 20 and 30 μm. The current-voltage (I-V) and current-power density (I-P) curves between 650 and 800°C are shown in Figures 8B–D. These corresponding values are displayed in Table 3. The maximum open-circuit voltage (OCV) of Cell-I, Cell-II, and Cell-III were 0.807, 0.898, and 0.836 V, respectively, which were all lower than the theoretical value of 1.04–1.1 V (Wang H. et al., 2019; Chen et al., 2020). In the high-temperature range, the OCV was further reduced. This was due to the partial reduction of Ce^4+^to Ce^3+^in the high-temperature reduction atmosphere, and the GDC electrolyte had a certain n-type conductivity, resulting in the internal short circuit of the cell and the decrease of OCV value (Bai et al., 2021; Han et al., 2021). At 650–800°C, the power densities of the three materials all followed the following order: PSFN_113_ < PSFN_214_ < PSFN_113-214_ (5:5), which corresponded to the above-mentioned R_P_ results. At 800°C, the maximum power density (PPD) of Cell-III (0.699 W cm^−2^) was 1.44 times that of Cell-I (0.485 W cm^−2^) and 1.24 times that of Cell-II (0.562 W cm^−2^). Cell performance was significantly enhanced. The PPD value of the PSFN_113-214_ (5:5) cathode was compared with the cathode performance reported in the literature, which further illustrated the superiority of its performance, as shown in Table 4.

**TABLE 3:** OCV (V) and PPD (W·cm^−2^) of Cell-I, Cell-II, and Cell-III in the range of 650–800°C.

Temperature (°C)	Cell-I		Cell-II		Cell-III	
	OCV	PPD	OCV	PPD	OCV	PPD
800	0.748	0.485	0.779	0.562	0.733	0.699
750	0.777	0.235	0.801	0.481	0.755	0.586
700	0.778	0.146	0.856	0.409	0.801	0.528
650	0.807	0.103	0.898	0.347	0.836	0.333

**TABLE 4:** Comparison table of PPD value of PSFN cathode and other cathode reported in literature.

Cathode	Cell configuration	PPD/(W·cm^−2^)	Temperature/°C	Reference
Ba_0.5_Sr_0.5_Fe_0.8_Cu_0.2_O_3-δ_	NiO-GDC|GDC|BSFC	0.51	700	Bai et al. (2021)
Pr_0.5_Ba_0.5_Fe_0.8_Ni_0.2_O_3-δ_	NiO-GDC|GDC|PBFN	0.52	700	Meng et al. (2021)
Pr_1.91_Ni_0.71_Cu_0.24_Ga_0.05_O_4_-Ba_0.5_La_0.5_CoO_3_	Ni-Fe|LSGM| PNCG-BLC	0.117	400	Xie et al. (2013)
La_0.6_Ca_0.4_Fe_0.8_Ni_0.2_O_3-δ_-Sm_0.2_Ce_0.8_O_1.9_	LCFN-30SDC/SDC/LCFN-30SDC	0.303	800	Ding et al. (2017)
PrLaNiO_4_-(La_0.75_Sr_0.2_Ba_0.05_)_0.175_Ce_0.825_O_1.891_	NiO-SDC|SDC| PLNO-LSBC	0.606	800	Chiu et al. (2017)
Pr_2_NiO_4_-Pr_0.2_Ce_0.8_O_1.9_	NiO-GDC|GDC|PNO-PCO	0.57	800	Chen et al. (2020)
Ce_0.8_Sm_0.2_O_2-δ_-La_0.25_Sr_0.75_Ti_1_O_3-δ_-Ni_0.8_Co_0.15_Al_0.05_LiO_2-δ_	Ni-LST-SDC-NCAL|LST-SDC-NCAL|LST-SDC-NCAL-Ni	0.222	550	Gao et al. (2020)
Pr_0.8_Sr_0.2_Fe_0.7_Ni_0.3_O_3-δ_-Pr_1.2_Sr_0.8_Fe_0.4_Ni_0.6_O_4+δ_ (5:5)	NiO-GDC|GDC|PSFN_113-214_ (5:5)	0.699	800	This work

**FIGURE 9:**
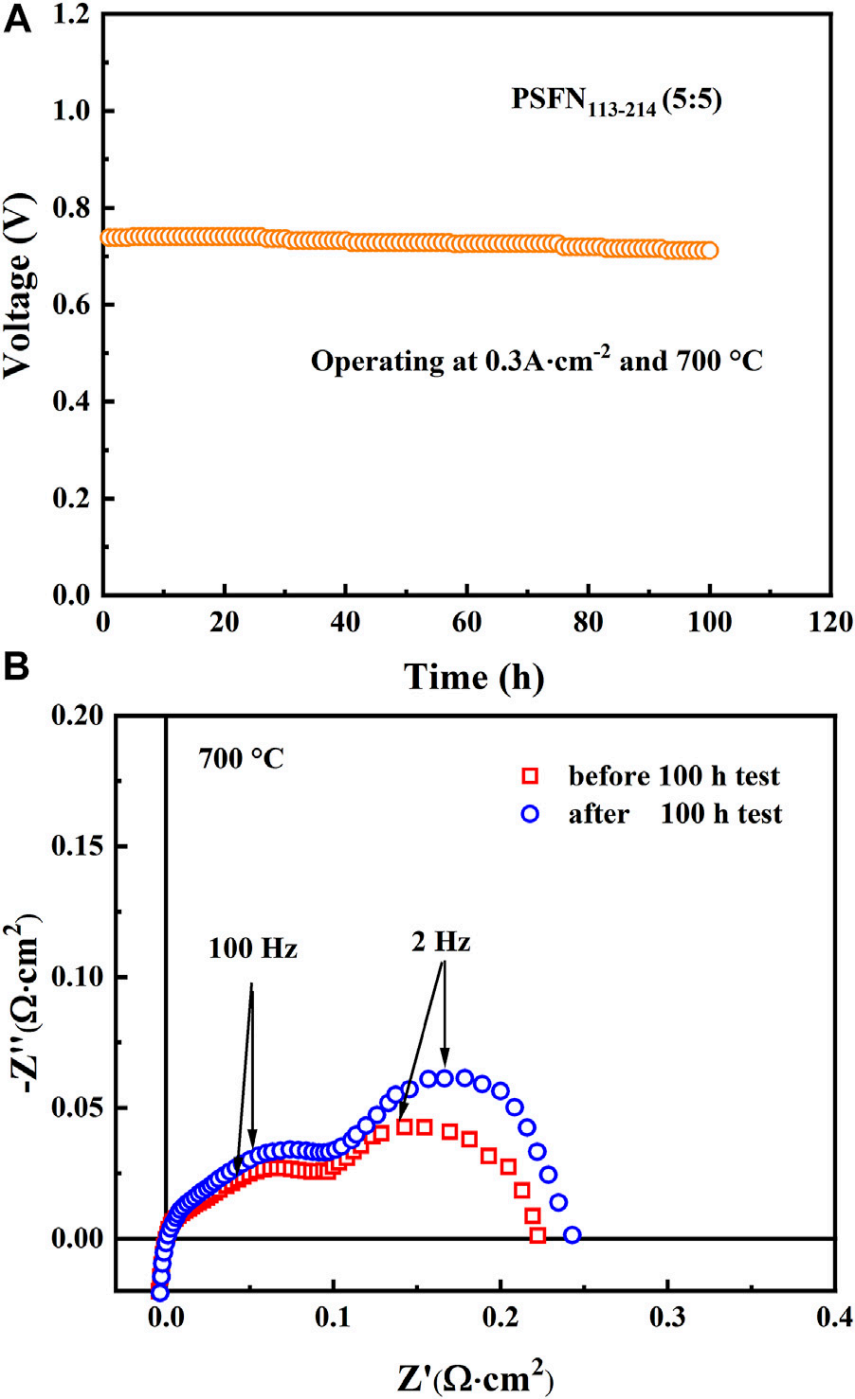
**(A)** The long-term stability test of a single cell constructed *via* PSFN_113-214_ (5:5) cathode **(B)** cross-sectional view of it.

**FIGURE 10:**
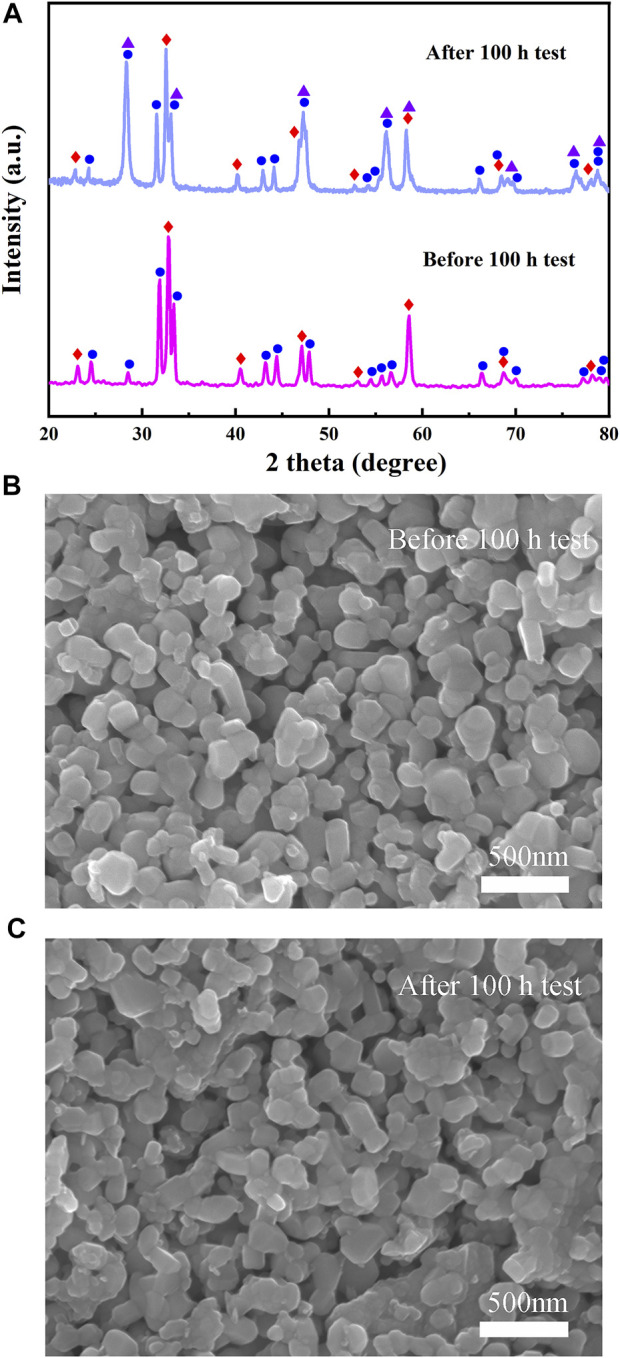
**(A)** XRD comparison pattern of PSFN_113-214_ (5:5) cathode before and after long-term stability test and SEM images of PSFN_113-214_ (5:5) cathode: **(B)** before long-term stability test; **(C)** after long-term stability test.

Furthermore, to study the stability of Cell-III, a 100-h long-term stability test was conducted under the conditions of 0.3 A cm^−2^ and 700°C, as shown in Figure 9A. These results showed that the initial voltage of Cell-III decreased from 0.738 to 0.712 V after 100 h of polarization, and the degradation rate of OCV was about 0.0352% h^−1^. Figure 9B shows the change in electrochemical impedance spectrum of cell-III before and after the stability test. Simultaneously, the XRD and SEM of PSFN_113-214_ (5:5) cathode after the long-term stability test were tested, as shown in Figure 10. The results showed that the XRD pattern only contained the characteristic peaks of cathode and electrolyte, and there was no impurity peak. And the cathode morphology had no obvious change. These results further proved that PSFN_113-214_ (5:5) could be used for long-term operation. PSFN_113-214_ (5:5) had good single-cell activity and stability, so it had broad application prospects in the intermediate temperature range. Afterward, cell activity can be improved via applying anode-supported single cells or diminishing the thickness of the electrolyte.

## Conclusion

Herein, new Pr_0.8_Sr_0.2_Fe_0.7_Ni_0.3_O_3−δ_-Pr_1.2_Sr_0.8_Fe_0.4_Ni_0.6_O_4+δ_ (PSFN_113-214_) cathode materials were prepared. The effect of the existence of heterointerfaces on the structures and properties of Pr_0.8_Sr_0.2_Fe_0.7_Ni_0.3_O_3-δ_ (PSFN_113_) and Pr_1.2_Sr_0.8_Fe_0.4_Ni_0.6_O_4+δ_ (PSFN_214_) was systematically studied. PSFN_113_ showed a good orthorhombic perovskite-type structure. PSFN_214_ showed a typical K_2_NiF_4_-type tetragonal structure. They were stable to each other and compatible with GDC electrolytes. Two single phases that make up the composite cathode were well mixed and tightly intertwined. When the heterocomposite cathode obtained by mixing at a mass ratio of 5:5 had the largest oxygen vacancy content (0.9836%) and specific surface area (5.1687 m^2^ g^−1^), the heterointerface was maximized. At 800°C, the R_P_ value of the heterocomposite cathode PSFN_113-214_ (5:5) was down to 0.029 Ω cm^2^ and the PPD for the corresponding single cell was as high as 0.699 W cm^−2^. The voltage decay rate was merely 0.0352% h^−1^ after 100 h. Therefore, PBFN_113-214_ (5:5) has broad application prospects.

## Data Availability

The original contributions presented in the study are included in the article/supplementary material, further inquiries can be directed to the corresponding authors.

## Nomenclature

SOFCs, solid oxide fuel cells; IT-SOFCs, intermediate-temperature solid oxide fuel cells; ORR, oxygen reduction reaction; O_lattice,_ lattice oxygen; O_surface_, surface oxygen; Sr_lattice_, lattice strontium; Sr_surface_, surface strontium; R_P_, polarization resistance; I-V, current-voltage; I-P, current-power density; OCV, open circuit voltage; PPD, maximum power density; XRD, X-ray diffraction; FT-IR, Fourier transform infrared spectroscopy; FE-SEM, field emission scanning electron microscope; XPS, X-ray photoelectron spectrometer; HR-TEM, high-resolution transmission electron microscope; EIS, electrochemical impedance spectroscopy; PSFN_113_, Pr_0.8_Sr_0.2_Fe_0.7_Ni_0.3_O_3-δ_; PSFN_214_, Pr_1.2_Sr_0.8_Fe_0.4_Ni_0.6_O_4+δ_; PSFN_113-214_ (4:6), samples with mass ratios of PSFN_113_ and PSFN_214_ of 4:6; PSFN_113-214_ (5:5), samples with mass ratios of PSFN_113_ and PSFN_214_ of 5:5; PSFN_113-214_ (6:4), samples with mass ratios of PSFN_113_ and PSFN_214_ of 6:4; GDC, Ce_0.8_Gd_0.2_O_1.9_; Cell-I, an electrolyte-supported NiO-GDC/GDC/PSFN_113_ single cell; Cell-II, an electrolyte-supported NiO-GDC/GDC/PSFN_214_ single cell; Cell-III, an electrolyte-supported NiO-GDC/GDC/PSFN_113-214_ (5:5) single cell

## References

[B1] Ahmad M. Z. Ahmad S. H. Chen R. S. Ismail A. F. Hazan R. Baharuddin N. A. (2022). Review on Recent Advancement in Cathode Material for Lower and Intermediate Temperature Solid Oxide Fuel Cells Application. Int. J. Hydrogen Energy 47, 1103–1120. 10.1016/j.ijhydene.2021.10.094

[B2] Ajaa A. Nab A. Mrs A. Ama B. (2020). Review of Composite Cathodes for Intermediate-Temperature Solid Oxide Fuel Cell Applications. Ceram. Int. 46 (15), 23314–23325. 10.1016/j.ceramint.2020.06.176

[B3] Bai J. Han Z. Lv B. Chen X. Zhu X. Zhou D. (2021). Preparation of 3D Structure High Performance Ba_0.5_Sr_0.5_Fe_0.8_Cu_0.2_O_3-δ_ Nanofiber SOFC Cathode Material by Low-Temperature Calcination Method. Int. J. Hydrogen Energy 46 (11), 8132–8142. 10.1016/j.ijhydene.2020.11.263

[B4] Bansod M. B. Khandale A. P. Kumar R. V. Bhoga S. S. (2018). Crystal Structure, Electrical and Electrochemical Properties of Cu Co-doped Pr_1.3_Sr_0.7_NiO_4+δ_ Mixed Ionic-Electronic Conductors (MIECs). Int. J. Hydrogen Energy 43 (1), 373–384. 10.1016/j.ijhydene.2017.11.005

[B5] Bhoga S. S. Khandale A. P. Pahune B. S. (2014). Investigation on Pr_2-*x* _Sr_ *x* _NiO_4+δ_ (*X* =0.3-1.0) Cathode Materials for Intermediate Temperature Solid Oxide Fuel Cell. Solid State Ionics 262, 340–344. 10.1016/j.ssi.2013.09.041

[B6] Chen X. Wang J. Liang Q. Sun X. Zhu X. Zhou D. (2020). Pr_2_NiO_4_-Pr_0.2_Ce_0.8_O_1.9_ Composite Cathode as a Potential Cathode Material for Intermediate Temperature Solid Oxide Fuel Cells. Solid State Sci. 100, 106108–106116. 10.1016/j.solidstatesciences.2019.106108

[B7] Chiu T.-W. Lin M.-X. Shih H.-Y. Hwang B.-y. Chang H.-Y. Wang Y.-M. (2017). Preparation and Performance of PrLaNiO_4_ and (La_0.75_Sr_0.2_Ba_0.05_)_0.175_Ce_0.825_O_1.891_ Composite Cathode Material by Solid State Reaction for IT-SOFCs. Ceram. Int. 43 (1), S700–S704. 10.1016/j.ceramint.2017.05.269

[B8] Ding X. Liu H. Gao Z. Hua G. Wang L. Ding L. (2017). La_0.6_Ca_0.4_Fe_0.8_Ni_0.2_O_3-δ_ -Sm_0.2_Ce_0.8_O_1.9_ Composites as Symmetrical Bi-electrodes for Solid Oxide Fuel Cells through Infiltration and *In-Situ* Exsolution. Int. J. Hydrogen Energy 42 (39), 24968–24977. 10.1016/j.ijhydene.2017.08.089

[B9] Dong Y. J. Han G. D. Choi H. R. Kim M. S. Choi H. J. Shim J. H. (2019). La_0.6_Sr_0.4_Co_0.2_Fe_0.8_O_3-δ_ Cathode Surface-Treated with La_2_NiO_4+δ_ by Aerosolassisted Chemical Vapor Deposition for High Performance Solid Oxide Fuel Cells. Ceram. Int. 45, 12366–12371. 10.1016/j.ceramint.2019.03.162

[B10] Gao J. Xu S. Akbar M. Xia C. Dong W. Liu C. (2021). Single Layer Low-Temperature SOFC Based on Ce_0.8_Sm_0.2_O_2-δ_-La_0.25_Sr_0.75_Ti_1_O_3-δ_-Ni_0.8_Co_0.15_Al_0.05_LiO_2-δ_ Composite Material. Int. J. Hydrogen Energy 46 (15), 9775–9781. 10.1016/j.ijhydene.2020.07.043

[B11] Ghamarinia M. Babaei A. Zamani C. (2020). Electrochemical Characterization of La_2_NiO_4_ -infiltrated La_0.6_Sr_0.4_Co_0.2_Fe_0.8_O_3-δ_ by Analysis of Distribution of Relaxation Times. Electrochimica Acta 353, 136520. 10.1016/j.electacta.2020.136520

[B12] Gilev A. R. Kiselev E. A. Cherepanov V. A. (2018). Oxygen Transport Phenomena in (La,Sr)_2_(Ni,Fe)O_4_ Materials. J. Mat. Chem. A 6 (13), 5304–5312. 10.1039/C7TA07162K

[B13] Giuliano A. Nicollet C. Fourcade S. Mauvy F. Carpanese M. P. Grenier J.-C. (2017). Influence of the Electrode/electrolyte Interface Structure on the Performance of Pr_0.8_Sr_0.2_Fe_0.7_Ni_0.3_O_3-δ_ as Solid Oxide Fuel Cell Cathode. Electrochimica Acta 236, 328–336. 10.1016/j.electacta.2017.03.179

[B14] Han Z. Bai J. Chen X. Zhu X. Zhou D. (2021). Novel Cobalt-free Pr_2_Ni_1-*x* _Nb_ *x* _O_4_ (*X*=0, 0.05, 0.10, and 0.15) Perovskite as the Cathode Material for IT-SOFC. Int. J. Hydrogen Energy 46 (21), 11894–11907. 10.1016/j.ijhydene.2021.01.045

[B15] Hashimoto S.-I. Kammer K. Larsen P. H. Poulsen F. W. Mogensen M. (2005). A Study of Pr_0.7_Sr_0.3_Fe_1-*x* _Ni_ *x* _O_3-δ_ as a Cathode Material for SOFCs with Intermediate Operating Temperature. Solid State Ionics 176 (11-12), 1013–1020. 10.1016/j.ssi.2004.09.010

[B16] Hou J. Wang Q. Li J. Lu Y. Wang L. Fu X.-Z. (2020). Rational Design of an *In-Situ* Co-assembly Nanocomposite Cathode La_0.5_Sr_1.5_MnO_4+δ_-La_0.5_Sr_0.5_MnO_3-δ_ for Lower-Temperature Proton-Conducting Solid Oxide Fuel Cells. J. Power Sources 466, 228240. 10.1016/j.jpowsour.2020.228240

[B17] Huan D. Zhang L. Zhu K. Li X. Shi N. Yang Y. (2021). Oxygen Vacancy-Engineered Cobalt-free Ruddlesden-Popper Cathode with Excellent CO2 Tolerance for Solid Oxide Fuel Cells. J. Power Sources 497, 229872–229881. 10.1016/j.jpowsour.2021.229872

[B18] Khoshkalam M. Tripković Ð. Tong X. Faghihi-Sani M. A. Chen M. Hendriksen P. V. (2020). Improving Oxygen Incorporation Rate on (La_0.6_Sr0.4)0._98_FeO_3-δ_ via Pr_2_Ni_1-x_Cu_x_O_4+δ_ Surface Decoration. J. Power Sources 457, 228035. 10.1016/j.jpowsour.2020.228035

[B19] Kim D. Lee K. T. (2021). Effect of Lanthanide (Ln=La, Nd, and Pr) Doping on Electrochemical Performance of Ln_2_NiO_4+δ_-YSZ Composite Cathodes for Solid Oxide Fuel Cells. Ceram. Int. 47 (2), 2493–2498. 10.1016/j.ceramint.2020.09.092

[B20] Kim S. J. Akbay T. Matsuda J. Takagaki A. Ishihara T. (2019). Strain Effects on Oxygen Reduction Activity of Pr2NiO4 Caused by Gold Bulk Dispersion for Low Temperature Solid Oxide Fuel Cells. ACS Appl. Energy Mat. 2 (2), 1210–1220. 10.1021/acsaem.8b01776

[B21] Kuzmin A. V. Lesnichyova A. S. Tropin E. S. Stroeva A. Y. Vorotnikov V. A. Solodyankina D. M. (2020). LaScO_3_-based Electrolyte for Protonic Ceramic Fuel cells:Influence of Sintering Additives on the Transport Properties and Electrochemical Performance. J. Power Sources 466, 228255–228264. 10.1016/j.jpowsour.2020.228255

[B22] Larramendi I. R. d. Antón R. L. Larramendi J. I. R. d. Baliteau S. Mauvy F. Grenier J. C. (2007). Structural and Electrical Properties of Thin Films of Pr_0.8_Sr_0.2_Fe_0.8_Ni_0.2_O_3-δ_ . J. Power Sources 169 (1), 35–39. 10.1016/j.jpowsour.2007.01.077

[B23] Lee D. Lee H. N. (2017). Controlling Oxygen Mobility in Ruddlesden-Popper Oxides. Materials 10 (4), 368–390. 10.3390/ma10040368 PMC5506909 28772732

[B24] Li F. Jiang L. Zeng R. Wang F. Xu Y. Huang Y. (2017). Hetero-structured La_0.5_Sr_0.5_CoO_3-δ_/LaSrCoO_4±δ_ Cathode with High Electro-Catalytic Activity for Solid-Oxide Fuel Cells. Int. J. Hydrogen Energy 42 (49), 29463–29471. 10.1016/j.ijhydene.2017.10.001

[B25] Li F. Xu Y. Cheng F. Yan Y. Xia S. Liu J. (2020). Composite Fibers of La_0.5_Sr_0.5_CoO_3-δ_-LaSrCoO_4±δ_ with High Catalytic Activity toward Oxygen Reduction. Ceram. Int. 46 (5), 6191–6198. 10.1016/j.ceramint.2019.11.086

[B26] Liu S. Yu B. Zhang W. Zhai Y. Chen J. (2016). Electrochemical Performance of Co-containing Mixed Oxides as Oxygen Electrode Materials for Intermediate-Temperature Solid Oxide Electrolysis Cells. Int. J. Hydrogen Energy 41 (36), 15952–15959. 10.1016/j.ijhydene.2016.05.077

[B27] Liu X. Yang Y. Ding Y. Chen Y. Gu Q. Tian D. (2017). Mo-doped Pr_0.6_Sr_0.4_Fe_0.8_Ni_0.2_O_3-δ_ as Potential Electrodes for Intermediate-Temperature Symmetrical Solid Oxide Fuel Cells. Electrochimica Acta 227, 33–40. 10.1016/j.electacta.2016.12.170

[B28] Meng Y. Zhang Q. Chen Z. Chen X. Zhou J. Zhu X. (2021). Novel Cobalt and Strontium-free Perovskite Pr_0.5_Ba_0.5_Fe_1-x_Ni_x_O_3-δ_ (X=0 and 0.2) as Cathode for Intermediate-Temperature Solid Oxide Fuel Cells. Ionics 27, 3951–3965. 10.1007/s11581-021-04148-0

[B29] Miao L. Hou J. Gong Z. Jin Z. Liu W. (2019). A High-Performance Cobalt-free Ruddlesden-Popper Phase Cathode La_1.2_Sr_0.8_Ni_0.6_Fe_0.4_O_4+δ_ for Low Temperature Proton-Conducting Solid Oxide Fuel Cells. Int. J. Hydrogen Energy 44 (14), 7531–7537. 10.1016/j.ijhydene.2019.01.255

[B30] Pinedo R. de Larramendi I. R. de Muro I. G. Insausti M. de Larramendi J. I. R. Arriortua M. I. (2011). Influence of Colloidal Templates on the Impedance Spectroscopic Behaviour of Pr_0.7_Sr_0.3_Fe_0.8_Ni_0.2_O_3_ for Solid Oxide Fuel Cell Applications. Solid State Ionics 192 (1), 235–240. 10.1016/j.ssi.2010.05.057

[B31] Wang H. Zhang X. Zhang W. Wei Z. Guan K. Meng J. (2019). Enhancing Catalysis Activity of La_0.6_Sr_0.4_Co_0.8_Fe_0.2_O_3-δ_ Cathode for Solid Oxide Fuel Cell by a Facile and Efficient Impregnation Process. Int. J. Hydrogen Energy 44 (26), 13757–13767. 10.1016/j.ijhydene.2019.03.184

[B32] Wang J. Chen X. Xie S. Chen L. Wang Y. Meng J. (2019). Bismuth Tungstate/neodymium-Doped Ceria Composite Electrolyte for Intermediate-Temperature Solid Oxide Fuel Cell: Sintering Aid and Composite Effect. J. power sources 428, 105–114. 10.1016/j.jpowsour.2019.04.105

[B33] Wang L. Wang P. Geng C. Cao H. Xu C. Cheng J. (2020). A Novel Core-Shell LSCF Perovskite Structured Electrocatalyst with Local Hetero-Interface for Solid Oxide Fuel Cells. Int. J. Hydrogen Energy 45 (20), 11824–11833. 10.1016/j.ijhydene.2020.02.130

[B34] Xie J. Ju Y.-W. Matsuka M. Ida S. Ishihara T. (2013). Synergy Effects of Pr_1.91_Ni_0.71_Cu_0.24_Ga_0.05_O_4_ and Ba_0.5_La_0.5_CoO_3_ Composite on Cathodic Activity for Intermediate Temperature Solid Oxide Fuel Cells. J. Power Sources 228, 229–236. 10.1016/j.jpowsour.2012.11.089

[B35] Yang J. Cheng J. Jiang Q. Wang Y. Wang R. Gao J. (2012). Preparation and Electrochemical Properties of Strontium Doped Pr 2 NiO 4 Cathode Materials for Intermediate-Temperature Solid Oxide Fuel Cells. Int. J. Hydrogen Energy 37 (2), 1746–1751. 10.1016/j.ijhydene.2011.09.146

[B36] Yang Y. Li R. Wu Y. Chu Y. Tian D. Lu X. (2020). Highly Active Self-Assembled Hybrid Catalyst with Multiphase Heterointerfaces to Accelerate Cathodic Oxygen Reduction of Intermediate-Temperature Solid Oxide Fuel Cells. Ceram. Int. 46 (7), 9661–9668. 10.1016/j.ceramint.2019.12.233

[B37] Yu X. Sui C. Ren R. Qiao J. Sun W. Wang Z. (2019). Construction of Heterointerfaces with Enhanced Oxygen Reduction Kinetics for Intermediate-Temperature Solid Oxide Fuel Cells. ACS Appl. Energy Mat. 3 (1), 447–455. 10.1021/acsaem.9b01701

[B38] Zhang L. Chen G. Dai R. Lv X. Yang D. Geng S. (2021). A Review of the Chemical Compatibility between Oxide Electrodes and Electrolytes in Solid Oxide Fuel Cells. J. Power Sources 492, 229630–229649. 10.1016/j.jpowsour.2021.229630

[B39] Zhao C. Li Y. Zhang W. Zheng Y. Lou X. Yu B. (2019). Heterointerface Engineering for Enhancing the Electrochemical Performance of Solid Oxide Cells. Energy Environ. Sci. 13, 53–85. 10.1039/C9EE02230A

[B40] Zheng Y. Li Y. Wu T. Zhao C. Zhang W. Zhu J. (2019). Controlling Crystal Orientation in Multilayered Heterostructures toward High Electro-Catalytic Activity for Oxygen Reduction Reaction. Nano Energy 62, 521–529. 10.1016/j.nanoen.2019.05.069

[B41] Zheng Y. Zhao C. Li Y. Zhang W. Wu T. Wang Z. (2020a). Directly Visualizing and Exploring Local Heterointerface with High Electro-Catalytic Activity. Nano Energy 78, 105236. 10.1016/j.nanoen.2020.105236

[B42] Zheng Y. Zhao C. Wu T. Li Y. Zhang W. Zhu J. (2020b). Enhanced Oxygen Reduction Kinetics by a Porous Heterostructured Cathode for Intermediate Temperature Solid Oxide Fuel Cells. Energy AI 2, 100027. 10.1016/j.egyai.2020.100027

